# Evolution of the PE_PGRS Proteins of Mycobacteria: Are All Equal or Are Some More Equal than Others?

**DOI:** 10.3390/biology14030247

**Published:** 2025-02-28

**Authors:** Bei Chen, Belmin Bajramović, Bastienne Vriesendorp, Herman Pieter Spaink

**Affiliations:** Institute of Biology, Leiden University, Einsteinweg 55, 2333 CC Leiden, The Netherlands; b.chen@liacs.leidenuniv.nl (B.C.); belminbajramovic@outlook.com (B.B.); b.vriesendorp@biology.leidenuniv.nl (B.V.)

**Keywords:** PE_PGRS, *Mycobacterium tuberculosis*, host immune response, AlphaFold3, phase separation

## Abstract

PE_PGRS domain proteins represent a family of proteins found in pathogenic and non-pathogenic mycobacteria such as *M. smegmatis*. This conserved family is characterized by two distinct regions denoted as the variable PGRS domain defined by glycine-rich repeats, and a PE domain consisting of two antiparallel alpha-helices. There are many indications that PE_PGRS proteins are involved in immunopathogenesis and virulence by evading or triggering the host immune response. However, there is not yet any information on their degree of specialization or redundancy. Computational analysis and structural annotation using AlphaFold3 combined with other tools reveals an exceptionally powerful and unprecedented ability to undergo phase separation by the PGRS domain. This suggests that PGRS’s glycine-rich, multivalent, low-complexity composition supports phase separation while adopting a structured conformation, contrary to the disordered nature typical of such domains. While previously never reported, the hypothesized role of PGRS in virulence indicates a novel window into the seemingly ubiquitous role of phase separation in cellular compartmentalization and molecular dynamics. This review aims to summarize the current understanding of the PE_PGRS family and its various biological roles in the context of bioinformatic analyses of some interesting representatives of *M. marinum* that are under control by host sterols. Based on the structural bioinformatics analysis, we discuss future approaches to uncover the mechanistic role of this intriguing family of mycobacterial proteins in both pathogenic and non-pathogenic mycobacteria.

## Simple Summary

Due to widespread disease caused by pathogenic mycobacteria such as *Mycobacterium tuberculosis*, many researchers have been looking for long-lasting strategies to combat these pathogens. In this review, we will discuss the conserved family of PE_PGRS proteins found in many pathogenic and non-pathogenic mycobacteria in order to summarize their enigmatic structural characteristics and putative functions. Then we will discuss the proposed role in host immunity of some apparently specialized gene paralogs in pathogenic mycobacteria as well as promising research perspectives offered by structural protein bioinformatics for developing new therapies against tuberculosis.

## 1. Introduction: Tuberculosis and PE_PGRS Protein Family

Tuberculosis (TB) is a disease primarily caused by the pathogen *M. tuberculosis*, a member belonging to the *M. tuberculosis* complex (MTB complex). According to the World Health Organization (WHO), tuberculosis remains the world’s deadliest infectious disease. In recent decades, many bacterial species outside the MTB complex, collectively known as nontuberculous mycobacteria (NTM), have emerged as significant causes of mortality. The social impact of diseases caused by NTM bacteria is likely to become even more threatening because, like *M. tuberculosis*, NTM either naturally possess or are developing high resistance against conventional antibiotics [1,2]. Therefore, the development of new antibiotics or host-directed therapies that can complement current therapies is increasingly urgent. In respect to the prospects of host-directed therapies, it is worth mentioning that pathogenic mycobacteria have in common that they can stay dormant for many years and persist in the body without any indication of illness. Over time, the pathogens have evolved and developed various strategies to resist host immune responses. For example, they have developed many mechanisms that allow them to hijack the process of phagosome–lysosome fusion, inhibit acidification of phagosomes, and suppress autophagy and the apoptosis pathways used by macrophages for the clearance of pathogens. In this way, mycobacteria can survive within host macrophages by evading or subverting immune effector functions [3,4]. Detailed studies of many potential virulence factors involved in these functions could lead to the identification of new bacterial targets for antibiotics, as well as targets of the host for the development of novel host-directed therapies [1,5,6,7,8,9,10,11].

With the advances in the genomic sequencing of mycobacteria, evolutionary insights into the origins of many conserved gene families have been obtained [12]. One early observation was the presence of a highly conserved protein domain in a very large protein family called the PE/PPE domain family, not observed outside the genus *Mycobacterium*. PE-domain proteins are characterized by the presence of a proline-glutamic acid (PE) motif at positions eight and nine within a highly conserved N-terminal domain, which consists of approximately 110 amino acids. Similarly, PPE proteins contain a proline-proline-glutamic acid (PPE) motif at positions seven to nine in a highly conserved N-terminal domain of about 180 amino acids. In MTB complex species, up to seven percent of the coding capacity is represented by members of these gene families [13]. They have been aptly termed a “molecular mantra” by Brennan and Delogu [14] and have been extensively reviewed in over 30 review articles over the last few decades, clearly pointing out their potential as targets for developing antibiotics or vaccines [7,15,16,17,18,19,20]. The *C*-terminal domains of both PE and PPE protein families are highly variable in size and amino acid sequence. These proteins are often encoded by genes with a high frequency of repetitive GC-rich sequences that differ in copy number between related genes [21]. In the species of the MTB complex, the PE domain family is numerically dominated by a major group of proteins members that contain numerous glycine-rich repeats (GGA-GGX repeats, where “X” stands for any of the other known amino acids), called the PE_PGRS subfamily (for polymorphic GC-rich repetitive sequence).

Recently, the predicted structures of some of the representatives of this protein family in *M. tuberculosis* have been analyzed by Berisio and Delogu using AlphaFold modelling and have been described as a molecular sail that was hypothesized to be dynamically bound to the mycobacterial outer membrane with one hydrophobic rim of the protein [22]. Their enigmatic structures, uniquely found in mycobacteria, have stimulated much research on their function and evolutionary origins, as reviewed in many recent publications [23,24]. Much evidence points to a function of various PE_PGRS proteins in interference with host immunity and involvement in macrophage apoptosis. Notably, it could interact with TLR receptors, leading to the release of pro-inflammatory cytokines, as well as anti-inflammatory cytokines. However, the structure-function relationships of PE_PGRS proteins remain poorly understood. In this review, we only briefly summarize the current knowledge on the PE_PGRS protein family and focus on possible new approaches that make increased use of improvements in AI tools such as AlphaFold3. In addition, we highlight the importance of the use of NTM mycobacterial and non-pathogenic mycobacteria to obtain new insights in functions of this protein family. For instance, *M. marinum*, which encodes over 148 genes of the PE_PGRS family [24], is closely related to *M. tuberculosis* and has been intensively used in the zebrafish models for studying tuberculosis [25,26]. Importantly, we point out that further studies of the expression of this gene family in response to host factors can lead to new understanding of their functions. It has already been shown that *M. marinum* genes are expressed specifically in the environment of granulomas [27]. Here, we present bioinformatic analyses of previously published data suggesting that an unrelated PE_PGRS protein of *M. marinum* is involved in the response to host sterols, such as cholesterol. We discuss these findings in the context of possible research strategies to better understand the function of the PE_PGRS protein family.

## 2. The PE_PGRS Domain Protein Family

### 2.1. Overview of the PE-Domain Protein Family

The PE domain protein family is usually classified into the following subfamilies: (i) the PE-only subfamily; (ii) the PE-unique subfamily; and (iii) the PE-Polymorphic GC-rich (PE-PGRS) subfamily. Among them, the PE-only subfamily domain is less than 100 amino acids in length and typically associated with a PPE protein to form a heterodimer [28]. The PE-unique subfamily is characterized by distinct amino acid sequences of varying lengths at the protein’s C-terminal end, while the PE_PGRS subfamily contains numerous glycine-rich sequences (GGA-GGX repeats) [28,29]. PE-domain encoding genes have been shown to be organized in operons that encode stable and functional protein complexes. For example, the crystal structures of the proteins encoded by *Rv2430c* and *Rv2431c* have been resolved to reveal that the most conserved regions of the PE and PPE proteins act as the interaction sites [30].

Notably, over 60% (65 genes) of the annotated PE proteins of *M. tuberculosis* belong to the PE_PGRS subfamily. However, some of these are pseudogenes or lack typical PE_PGRS features, indicating that only 51 PE_PGRS proteins are potentially functional in the MTB complex [31]. The PE domain proteins are intrinsically disordered, redundant, and potentially antigenic in nature [16,31]. Despite the reductive genomic evolution of mycobacteria, this family of genes has expanded throughout evolution. In particular, the observation that this family has greatly expanded in pathogenic strains of the genus *Mycobacterium*, such as *M. tuberculosis*, *M. marinum*, and *M. bovis*, indicates its importance in disease pathogenesis. These proteins likely serve multiple functions that enhance the virulence of *M. tuberculosis*, primarily by modulating immune responses and affecting immune-mediated clearance of the pathogen. Genomic sequencing of multiple mycobacterial strains has revealed that all pathogenic mycobacteria express various PE/PPE-domain proteins likely involved in their virulence. Non-pathogenic strains of the *Mycobacterium* genus, such as *M. smegmatis*, also contain genes encoding PE/PPE-domain proteins, which are found within *ESX6* (Early Secreted Antigenic Target of 6 kDa-*ESAT-6 System*) gene clusters [32]. However, outside the genus *Mycobacterium*, there are no reported proteins that contain a recognizable PE/PPE domain [19].

**Figure 1:**
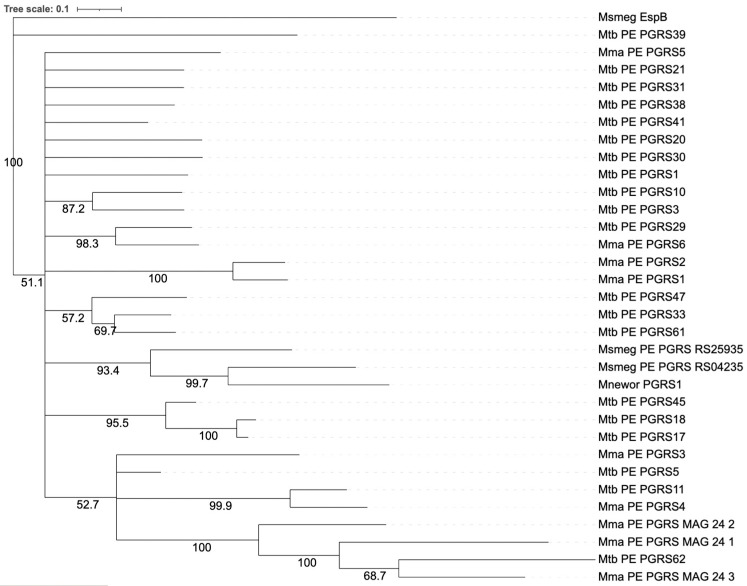
Neighbor-joining tree of the selection of PE_PGRS proteins with EspB of *M. smegmatis* included as an outgroup. The tree was based on the MUSCLE alignment and constructed by the neighbor-joining algorithm as implemented in Geneious Prime 2023.2.1 (https://www.geneious.com (accessed on 2 December 2024)). Branch numbers refer to bootstrap values (percentages) based on 1000 replicates. The protein sequences used for constructing this tree are given in Supplemental Table S1 in FASTA format. *Mnewor* is an abbreviation for *M. neworleansens*.

Over the past few years, many researchers have investigated the domain structures of this protein family, seeking to understand the relationship between their structure and function in host cells [16,33,34]. However, considering that there are so many different PE/PPE domain proteins in various mycobacteria, many difficulties are posed in the further exploration of their functions. The expansion of the PE/PPE gene families has been linked to the evolution of the *ESAT-6* secretion system, which is implicated in the secretion of heterodimers to the cell surface [32,35]. The cell surface localization of this protein family is expected to play a crucial role in the virulence and pathogenesis of mycobacteria [36,37]. In this review, we analyze the structures of various members of the PE_PGRS subfamily listed in Figure 1 to identify potential specialized functions.

### 2.2. Overview of the PE_PGRS Proteins

PE_PGRS proteins represent a large family of proteins typical of pathogenic mycobacteria whose members are characterized by an N-terminal PE domain. The highly conserved PE domain has been proposed to be responsible for protein translocation to the mycobacterial outer membrane and the variable PGRS domain is thought to be responsible for interactions with host components [38]. Its highly repetitive glycine-rich repeats show an analogy with the glycine-rich domain of well-known substrates of the ESX secretion machinery, notably the EspB (ESX-1 secretion-associated protein B) protein [33]. Therefore, a possible role of chaperone proteins from the EspG (ESX-1 secretion-associated protein G) family in secretions of PE_PGRS proteins could be considered [39]. As the major subclass of PE proteins, the PE_PGRS subfamily is present in a multitude of pathogenic strain of the genus *Mycobacterium* such as *M. tuberculosis* and other members of the MTB complex, *M. marinum*, and *M. ulcerans*, as well as several other related species. In all of them, a remarkable genetic homogeneity with few single nucleotide polymorphisms in some particular homologs can be identified within the various strains. Future analysis of the genome of *M. canetti*, which is thought to represent the closest living relative of the progenitor of the MTB complex, could reveal more details on the recent evolution of the PE_PGRS genes [39]. The highly preserved homologs in many pathogenic mycobacteria indicate that PE_PGRS proteins play an important role in mycobacterial pathogenesis [32].

However, our own bioinformatic investigation reveals that no identifiable genes encoding PE_PGRS domain proteins can be found in the genome of various pathogenic NTM strains such as *M. avium* and *M. abscessus*. Since nucleotide sequences of PE_PGRS genes often contain mistakes in the high GC regions of the genes and BLAST+ 2.16.0 searches with the highly repetitive PGRS domains are not completely trustworthy, we also manually inspected the genetic repertoire of some representatives of these species, but without success.

Surprisingly, we found that in many non-pathogenic mycobacterial strains such as *M. smegmatis* and related *Mycolicibacterium* species, we can identify proteins with a clear PGRS domain. AlphaFold3 predictions, as discussed below, show that the *M. smegmatis* model strain MC2 encodes two proteins that clearly display a typical PGRS structure that typifies this protein domain. Their structures clearly demonstrate the typical sail-like structure as reported for *M. tuberculosis* PGRS domains by Berisio and Delogu [24]. For comparison, we compared their predicted primary structures with the *M. tuberculosis* PE_PGRS proteins that have been studied for biological function, as discussed below (Figure 1). Unpublished RNAseq data from our laboratory show that these two *M. smegmatis* genes are expressed in standard growth medium. However, manual inspection of these genes in the reference genome shows that the annotation of the N-terminus of these genes is questionable and, therefore, the identity of a PE domain connected to the PGRS domain needs experimental confirmation. Since it has been argued that *M. smegmatis* cannot secrete heterologously expressed PE_PGRS proteins, their cellular localization remains disputable. Since several studies provide evidence of the expression of PE_PGRS proteins in NTM bacteria such as *M. smegmatis*, there is a great need to investigate the secretion of heterologously expressed PE_PGRS proteins in more detail.

### 2.3. The Subcellular Localization of PE_PGRS Proteins

Despite the abundance of PE_PGRS-coding genes in many mycobacterial genomes, their precise roles and functions in bacterial pathogenesis remain unknown. It is known that many *M. tuberculosis* PE_PGRS proteins are membrane attached and localize to the cell surface (PE_PGRS11/17/33/62), where they are involved in host–pathogen interactions [40,41]. Additionally, several PE_PGRS proteins have been identified as cell surface components that influence cellular architecture, colony morphology, and interactions with other cells [14,42,43].

Further evidence for the surface localization of the PE_PGRS proteins focuses on their amino acid composition as well as sequences. For example, Copin et al. [44] found that the proline-glutamic acid (PE) domain is responsible for the cellular localization of these proteins on bacterial cells. The glycine-rich motif repeats in the PGRS domain could form a Ca^2+^-binding structure, a parallel β-roll, or a parallel β-helix structure, which is also typical of a calcium-binding protein [45]. PE_PGRS proteins play a crucial role in *M. tuberculosis* virulence, particularly during the chronic phase of infection. During this phase, PE_PGRS proteins accumulate in granulomas where they can promote inflammation by directly interacting with Toll-like receptors (TLRs) through the PGRS domain [46].

For instance, PE_PGRS33, encoded by *Rv1818c*, is the best-studied protein of the PE_PGRS family. Not only is the protein surface-exposed and could influence bacterial cell structure, but it is also able to interact with TLR2 to promote cell death and inflammation. Notably, proper localization of PE_PGRS33 on the mycobacterial surface is essential for TLR2 pathway activation [47]. Additionally, PE_PGRS33 was previously observed to play a role in humoral response, with the PGRS domain as the main protein responsible for antibody generation [48]. Moreover, the anti-PE PGRS33 humoral response induced by immunizing C57BL/6 mice with native PE_PGRS33, but not with the 1818 PE_PGRS DNA vaccine, interfered with the pathogenic features associated with the infection of C57BL/6 mice with *M. smegmatis* expressing PGRS33 [49,50], suggesting that inhibition of the proinflammatory activities of PE_PGRS33 may impact the course of *M. tuberculosis* infection [51].

Importantly, recent work has found that the *ESX5* system, which is a type VII secretion system specialized in mycobacterial protein secretion, is important for PE_PGRS protein secretion [52]. As for the secretion of other PE domain-containing proteins, this might involve the function of EspG. However, as mentioned above, strains of the *M. avium* complex that possess a functional *ESX5* system do not encode any PE_PGRS proteins, indicating that the *ESX5* system also has other non-redundant functions. [53] In many cases, this PE domain is fused to large C-terminal domains that are not involved in the secretion process. A large group of the *pe* genes (more than 60 in *M. tuberculosis* and more than 130 in *M. marinum*) contain polymorphic GC-rich sequences (PGRS), which encode glycine-rich repeats that are postulated to play a role in immune evasion [54]. The absence of PGRS domain proteins in the *M. avium* complex seems to be correlated with the fact that this large mycobacterial group has no *ESX1* secretion system. Secretion of PE_PGRS is reportedly dysfunctional in mutants in the *ppe38* gene [55,56]. It has also been noted in *M. tuberculosis* that the loss of function of secretion of PE_PGRS proteins can lead to increased virulence of *M. tuberculosis* in the later stages of tuberculosis disease progression [55]. The latter observation can be linked to the finding that an *M. marinum* mutant with a disrupted *espG5* gene, putatively involved in PE_PGRS protein secretion, is hypervirulent during infection in zebrafish, but only when adaptive immunity has developed in adult zebrafish. Because of these findings, it has been suggested that PE_PGRS proteins might serve other functions in *M. tuberculosis* unrelated to pathogenicity.

Notwithstanding this critical observation, the fact that the PGRS domain is targeted by the humoral immunity in TB patients, coupled with the polymorphic nature of some PE_PGRS proteins, indicates that these proteins play important roles in immunity and pathogenicity. Therefore, we discuss their potential immune roles in host cells to explore their specific functions.

## 3. The Function of PE_PGRS Protein in Host Immune Responses

### 3.1. PE_PGRS Proteins Can Interact with the TLR Signaling Pathway

TLRs, as pattern recognition receptors, could recognize pathogen-associated molecular patterns found on mycobacteria or mycobacterial cell wall components, thereby leading to intracellular signaling events, such as the activation of innate immune cells, the secretion of inflammatory cytokines, and the initiation of adaptive immune responses [57,58,59,60,61,62]. For example, PE_PGRS33 proteins, located on the mycobacterial cell wall and membrane, interact with TLR2 and trigger the TLR2 signaling pathway [51].

**Figure 2:**
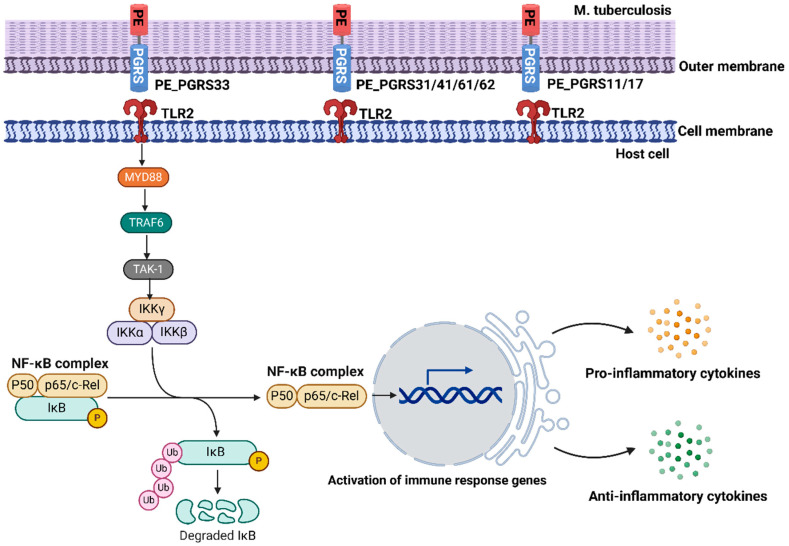
A brief overview of PE_PGRS protein activation with TLR2 in the host cell. Both PE_PGRS proteins and TLR2 are located on the membranes. The interaction of PE_PGRS with TLR2 could activate the pathway of NF-κB, leading to the production of cytokines involved in the immune responses. This figure is made by Biorender.

As described in Figure 2, after the interaction between PE_PGRS proteins and TLR2, its associated adaptor protein, Myeloid Differentiation Primary Response 88 (MYD88) is activated to recruit TNF Receptor-Associated Factor 6 (TRAF6). Activated TRAF6 then triggers the activity of TGF-β-activated kinase 1 (TAK1), which stimulates the activation of the inhibitor of nuclear factor kappa-B kinase complex (IKKα, IKKβ, and IKKγ, also known as IKK or NEMO). Furthermore, the IKK complex mediates the nuclear translocation of Nuclear Factor kappa-light-chain-enhancer of activated B cells (NF-κB). In turn, this results in the production of pro-inflammatory as well as anti-inflammatory cytokines. These factors could control inflammation responses and modulate cell survival. For example, at the early infection stage, TLR2 enhances the entrance of *M. tuberculosis* bacteria into macrophages by binding PE_PGR33, which is a typical mycobacterial protein from *M. tuberculosis* [51]. The binding of TLR2 and PE_PGR33 can activate macrophages by inducing the expression of TNF-α and some other pro-inflammatory cytokines [63,64].

There is a specialized secretion system classified as type VII secretion system, which is essential for the virulence of different pathogenic mycobacteria, such as *M. tuberculosis* and *M. marinum* [65,66]. This system is thought to be responsible for the transport of all extracellular proline-glutamic acid proteins encoded by the PE_PGRS protein detectable by Western blot analysis. It is also able to, independently from PE_PGRS proteins, to activate macrophage-mediated cytokine responses through TLR signaling including through TLR2 and TLR4. The fact that PE_PGRS proteins could activate TLR signaling pathways indicates that it can alter expression profiles of relevant inflammatory cytokines in a cell-specific manner, dependent on their secretion.

### 3.2. PE_PGRS Proteins Can Alter Profiles of Inflammatory Cytokines Within Macrophages

So far, many PE_PGRS proteins have been observed to modulate macrophage functions by altering pro- or anti-inflammatory responses. For example, Talarico et al. found increased secretion of IL-10 and reduced nitric oxide (NO) production by macrophages infected with *M. smegmatis* expressing PE_PGRS33 of *M. tuberculosis* [67]. Yeruva et al. concluded that *M. smegmatis* strains expressing PE_PGRS33/61 showed upregulation of IL-10 when regulated cytokines release and induces host cell death [68]. Deng et al. demonstrated that recombinant *M. smegmatis* expressing PE_PGRS41 downregulates macrophage inflammatory factors such as TNF-α, IL-1β, and IL-6, while upregulating the expression of the anti-inflammatory factor such as IL-10 [69]. And Kim et al. found that PE_PGRS38 downregulated the pro-inflammatory cytokines in BMDM and 293T cell lines [70].

Significantly, Huang et al. have shown that the expression of PE_PGRS62 in *M. smegmatis* decreased pro-inflammatory cytokine mRNA levels such as IL-1β and IL-6 in macrophages, and also reduced phagosome maturation and increased inducible nitric oxide synthase (iNOS) expression [71,72]. Long et al. found that PE_PGRS62 can disturb cytokine profiles of macrophages and further inhibit endoplasmic reticulum stress response (ERS)-mediated cell apoptosis, thus resulting in enhanced survival of non-pathogenic *M. smegmatis* within macrophages [73].

Additionally, one recent work showed that PE_PGRS31 promoted mycobacterial survival in macrophages by inhibiting the secretion of TNF-α through the TLR4-MyD88-NF-κB-TNF-α signaling axis via S100A9 [74]. Above all, many types of PE_PGRS proteins can alter the profiles of cytokines in macrophages through different pathways, which means they could play an important role in the host cells.

### 3.3. PE_PGRS Proteins Can Induce Apoptosis of Macrophages Through Modulation of Host Cell Death

It is well known that macrophages are the first line in defending against infection by *M. tuberculosis*. Pathogens enter macrophages via different receptor molecules and lead to apoptosis, which is used by the host to eliminate the intracellular bacteria. Inhibition of macrophage apoptosis could help *M. tuberculosis* to evade the priming of cytotoxic T cells by suppressing antigen presentation, escaping bacterial effects of apoptosis, and preserving a favorable cell environment for growth and persistence of mycobacteria. There are several publications that show that PE_PGRS proteins are involved in modulating host cell death to promote mycobacterial survival in macrophages [9,40,75].

For example, Basu et al. showed that recombinant PE_PGRS33 protein, as well as an *M. smegmatis* strain overexpressing PE_PGRS33, can induce apoptosis in RAW 264.7 cells [40]. Singh et al. found that MMAR_0242, an important PE_PGRS protein of *M. marinum*, can reduce cytotoxic cell death [76]. Deng et al. found that *M. smegmatis* expressing PE_PGRS41 recombinant, compared to the control, inhibited macrophage apoptosis via the caspase-dependent pathway [69]. Yang et al. showed that the overexpression of PE_PGRS18 markedly decreased *M. smegmatis* infection-induced apoptosis in THP-1 cells (a cell line derived from human monocytes) compared to Ms_Vec-infected THP-1 cells [77].

Additionally, Strong et al. demonstrated that RAW 264.7 macrophages infected with the PE_PGRS47 and PE_PGRS20 deletion mutants showed enhanced autophagy induction, attenuated growth, and a significant increase in CD^4+^ T cell responses to MHC class II presented antigens [11]. Xu et al. recently found that *M. smegmatis* with an overexpression of PE_PGRS45 could enhance bacterial viability under stress in vitro and cell survival in macrophages [78]. In conclusion, PE_PGRS proteins have been demonstrated to have diverse functions, including in macrophages, even though the specific pathways need to be elucidated further.

## 4. Specialization of PE_PGRS Proteins: Are All Equal or Are Some More Equal to Others

Previous studies have shown that some PE/PPE genes are polymorphic, a finding that suggests involvement in antigenic variation. McEvoy et al. used comparative sequence analysis of 18 publicly available MTB complex whole genome sequences and performed alignments on 33 PE (excluding PE_PGRS) and 66 PPE genes in order to detect the frequency and nature of genetic variation [79]. In this way, they found that while both PE and PPE genes had approximately threefold higher mutation rates than non-PE/PPE genes, no selective constraints were present in general, although their study excluded the PE_PGRS subset [23]. This has led to the current notion that PE_PGRS diversity is not driven by antigenic pressure [23]. It has been noted that PE_PGRS proteins contain only a few epitopes [62]. Of the 1649 known epitopes of *M. tuberculosis*, three are located in the PGRS domain of PE_PGRS proteins [52]. It therefore appears that there is antigenic pressure on a few of the PE_PGRS genes, while others seem to evolve neutrally. For instance, selection pressure is indicated for PE_PGRS33, one of the best-studied PE_PGRS proteins described above, where polymorphisms correlate with differences in pathogenicity of *M. tuberculosis* strains in patients [80]. This suggests that the group of PE_PGRS proteins may not be homogenous and that particular genes or subgroups of PE_PGRS proteins need to be examined for their biological functions from a perspective of infection-stage-specific expression [81,82]. With this in mind, it is of particular importance that the groundbreaking work of Ramakrishnan et al., in their establishment of the zebrafish larval *M. marinum* infection model, already identified three PE_PGRS genes that are specifically upregulated in granulomas [30]. These genes, known as membrane-associated genes (*mag23-1*, *mag24-2*, and *mag24-3*), encode PE_PGRS proteins that are highly similar to several *M. tuberculosis* PE_PGRS proteins, such as PE_PGRS62 (Figure 1). AlphaFold analysis shows that these proteins have a typical molecular sail structure, as observed in other PE_PGRS proteins (Supplemental Figure S2).

**Figure 3:**
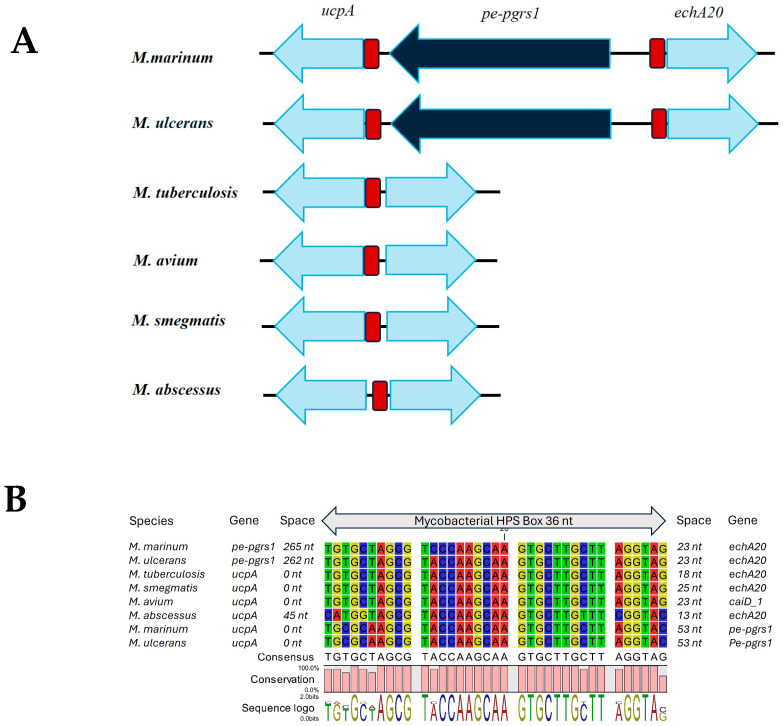
Genetic organization of the *M. marinum PE_PGRS1* gene compared to other mycobacteria. Panel (**A**): The orientation of the orthologs of the *PE_PGRS1* gene from *M. marinum* strain and the two neighboring genes. The gene preceding *PE_PGRS1* has been called *ucpA* based on the annotation of its ortholog in *M. avium*. The ortholog of this gene in *M. tuberculosis* has been assigned Rv3549c and its transcription was shown to be induced by. Orthologs of the *M. marinum* echA20 gene have been annotated as such in many other mycobacteria, but is annotated as *CaiD_1* in *M. avium*. The *PE_PGRS1* gene of *M. marinum* has two copies of very similar orthologues in a large group of related bacteria including *M. ulcerans*, as shown in the neighbor-joining tree and fast minimum evolution tree generated by fast minimum evolution settings at NCBI: https://blast.ncbi.nlm.nih.gov/Blast.cgi (accessed on 6 December 2024) shown in Figure 1 and Supplemental Figure S1. The *PE_PGRS1* genes of *M. marinum* and *M. ulcerans* are surrounded by two highly preserved sequence boxes shown in panel (**B**). Panel (**B**): Alignment of the highly preserved sequence (HPS) boxes surrounding *PE_PGRS1* of *M. marinum* and *M. ulcerans*, compared to the HPS boxes in between the orthologues of *ucpA* and *echA20*. A BLAST search (word size 16) identified 1087 hits with highly similar sequences in genomes of strains of the genus *Mycobacterium* (Supplemental Table S2). An additional 132 hits were found with a BLAST+ 2.16.0 search of the *M. abscessus* HPS box (Supplemental Table S2). The sequence logo is based only on the sequences depicted in panel (**B**).

In addition, our bioinformatic analysis of the gene clusters in *M. marinum* that are involved in host sterol responses gives a surprising clue to the importance of one PE_PGRS protein that has previously not been described. The comparative genome analysis shown in Figure 3 identified a very peculiar arrangement of one particular pair of operons in *M. marinum* that encode well-described enzymatic functions in cholesterol catabolism that are conserved in various mycobacteria. Part of the two operons shown in Figure 3 shows the highly conserved genes *Rv3549c*/*ucpA* and *echA20* that encode enzymes that are thought to be involved in breakdown of the C and D ring of cholesterol [83,84]. These genes, which are regulated by the tetracycline repressor family protein (TetR) KstR-like regulator 2 (KstR2), are not only conserved in all known mycobacteria, but are also organized in a remarkably similar operon structure across all known mycobacteria, except for one group closely related to *M. marinum*, including *M. ulcerans*, *M. liflandii*, and *M. pseudoshottsii* [84].

In all classified members of these bacterial species there is an insertion of an extremely highly preserved PE_PGRS gene that we have called *M. marinum PE_PGRS1* (Figure 1). What makes this locus even more remarkable is that, as shown in Figure 3, it is adjacent to a remarkably preserved non-coding sequence in mycobacterial genomes that we have called the mycobacterial HPS box (for highly preserved sequence). This sequence box is so extremely similar in all mycobacteria that it can even be used with BLAST searches to identify all known genomes of mycobacterial species (legend to Figure 3, Supplemental Table S2). However, it cannot be found with BLAST searches in bacteria not belonging the genus *Mycobacterium*. Since this HPS box contains a highly conserved consensus binding-sequence for the KstR2 protein, it can reasonably be hypothesized to be an important regulatory element for responses to host sterols (hence, the name HPS box could also stand for “highly preserved sterol response”). In support of this hypothesis, we have recently shown using RNAseq studies that the *PE_PGRS1* gene of *M. marinum* is indeed induced by the presence of host sterols such as cholesterol and bile acids in the growth medium (Herman P. Spaink, personal communication). Our laboratory is currently active in studying the function of the *M. marinum* PE_PGRS1 protein and comparing it to a very similar homolog encoded by all the *M. marinum* and *M. ulcerans* genomes that is not preceded by a mycobacterial HPS box, which we have called *M. marinum PE_PGRS2* (Figure 3).

## 5. Structure Function Relationships of PE_PGRS Proteins

**Figure 4:**
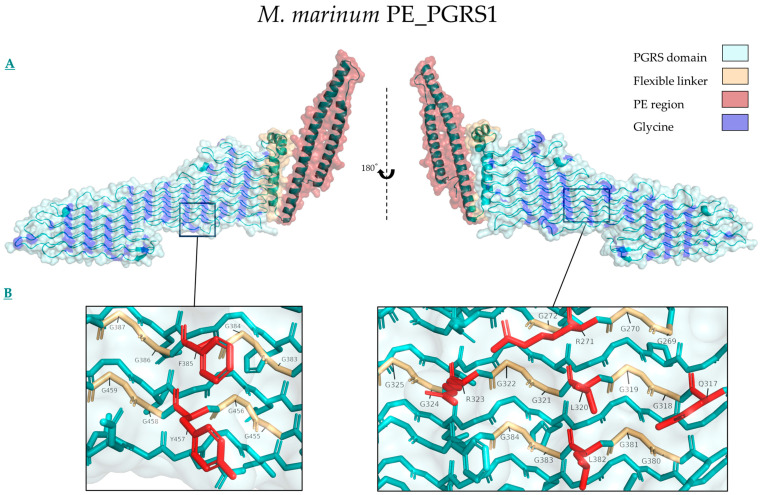
Predicted structure of *M. marinum* PE_PGRS1 made using AlphaFold3 on AlphaFold Server and annotated in PyMol. Prediction values are given in Supplemental Figure S5A. (**A**): The surface and ribbon representation show the different domains of the protein through having color-coded the different domains with red for the PE region, yellow for the flexible linker between PE and PGRS, cyan for the PGRS domain, and blue for the glycine distribution in the PGRS domain. (**B**): The inset shows the repetitive motif of double glycine flanking of reactive residues, with a focus on F385 and Y457 on the left, and R271, R323, L320, Q317, and L382 on the right.

Structural analysis using AlphaFold3 models of PGRS family proteins highlights a distinct, non-stochastic distribution of glycine residues in the PGRS domain [85] (Figure 4A). This repetitive pattern seems to be conserved even among distantly related homologs, although the PGII sandwich-stacking is oriented vertically in *M. marinum* PE_PGRS1 and horizontally in *M. tuberculosis* PE_PGRS1, *M. smegmatis* PE_PGRS_RS04235 and *RS25935*, and *M. neworleansense* PE_PGRS1 (Supplemental Figure S2). Despite the variation in stacking, the physico-chemical interface of double glycine-flanked reactive residues remains preserved. The reactive residues identified contain polar, charged, or aromatic sidechains, allowing the PGRS domain to be flexible due to the glycine distribution while enabling interaction through large protruding reactive sidechains on either side of the protein (Figure 4B).

**Figure 5:**
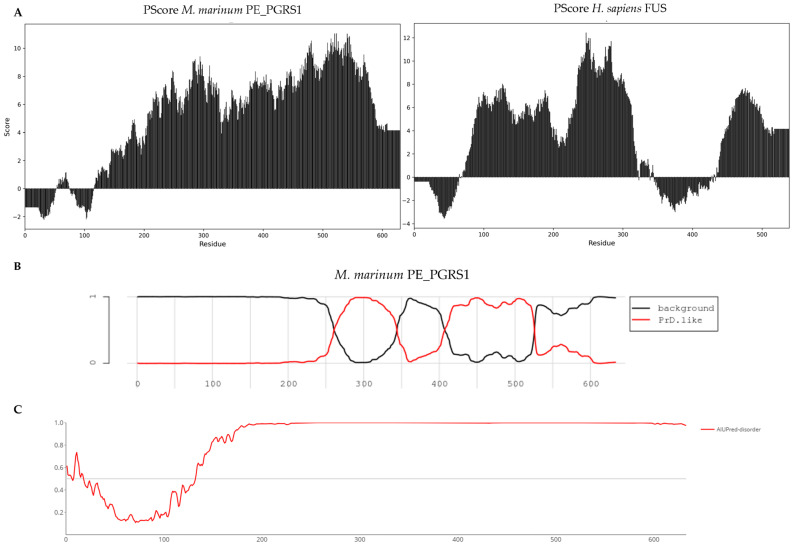
Sequence-based phase separation predictions using the PLAAC and PScore algorithms plotted using Python 3.9.0. (**A**): Bar plots show the PScore per residue for proteins *M. marinum* PE_PGRS1 and human FUS protein, with the PScore on the Y-axis and residue numbers on the X-axis. (**B**): PLAAC-generated plots predicting the presence of two prion-like regions (PrD-like) in red. (**C**): PLAAC-generated plot showing the predicted protein disorder based on the IUPRED3 algorithm.

The exceptional length of the glycine-rich low-complexity domain in *M. marinum* PE_PGRS1 suggests a strong potential for phase separation based on sequence similarity with previously studied phase-separating proteins (Figure 5A) [86,87,88,89]. This property supports emergent molecular behaviour linked to the spatiotemporal localization of biomolecules into membrane less organelles, known as biomolecular condensates [90]. These liquid-to-solid phase droplets perform diverse functions, such as selectively sequestering proteins or mRNA for protection, degradation, biochemical reactions, or environmental sensing. This presents an intriguing opportunity to explore the broad potential of PE_PGRS function in cellular processes. Sequence-based analysis using the PLAAC algorithm predicts two prion-like domains in PE_PGRS1—key elements of phase separation in proteins—indicating an ability to respond to environmental factors that modulate phase separation (Figure 5B) [91]. The same program predicts a high degree of disorder in the PGRS domain, contrasting with AlphaFold’s confident structural predictions.

Furthermore, the PScore prediction suggests that PE_PGRS1 has phase separation propensities even greater than that of the human FUS protein, responsible for forming nuclear paraspeckles (Figure 5A) [87]. This human protein was chosen because it is exceptionally well-studied in its structural properties related to phase separation, unparalleled compared to any bacterial phase separating protein of which few are known. Proteins with such high phase separation propensity typically act as scaffolds to recruit client proteins or other biomolecules [92]. Within this framework, the broader PGRS family may serve as nucleation factors in different cellular processes while potentially playing a role in virulence. In a similar mechanism, chaperones may modulate PE_PGRS protein phase-separating function by localization. Validation of such theories would require imaging studies using fluorescence recovery after photo-bleaching of fluorescently labelled PGRS proteins in vivo and in vitro phase separation assays to confirm whether PGRS proteins form condensate-like droplets. Follow-up studies could explore potential redundancy or functional specialization by tagging multiple PGRS proteins with different fluorescent markers and performing proximity labelling to analyze the PGRS interactome and condensate content.

**Figure 6:**
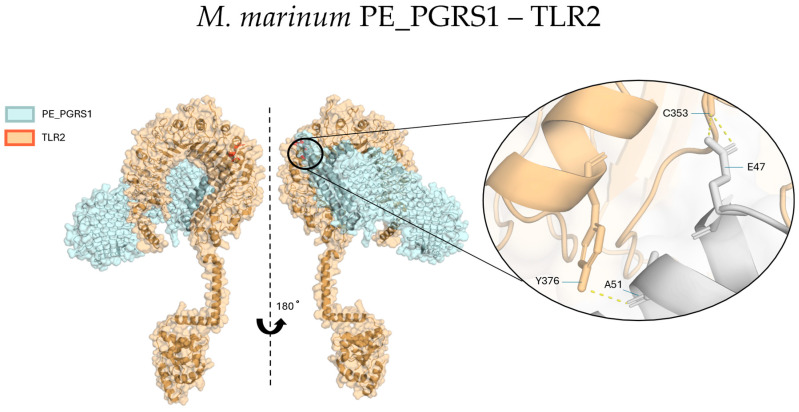
Predicted interaction between *M. marinum* PE_PGRS1 and *D. rerio* TLR2, made using AlphaFold3 on AlphaFold Server and the “find contacts” action in Pymol. The two proteins have been colored cyan and orange, respectively. The interaction interface is strongest and most stable at the PE region, with the inset showing in more detail the two identified polar contacts at PE_PGRS1 residues E47 and A51. Prediction values are shown in Supplemental Figure S4.

One impactful possibility is that *M. marinum* PE_PGRS1 phase separation contributes to virulence through interaction with TLR2. Our AlphaFold3 analysis provides detailed modelling of this interaction, as well as the previously reported interaction between *M. tuberculosis* PE_PGRS33 and TLR2, surpassing the description by Berisio and Delogu [24], and shows that the strongest, most stable binding occurs in the PE region, leaving the PGRS domain free to mediate phase separation and condensate localization at the receptor (Figure 6, Supplemental Figure S4). Combining condensate functions like sequestration and stabilization with TLR2 endocytosis suggests an exciting theory: PE_PGRS condensates may trigger TLR2 activation, introducing condensate-associated virulence factors into host cells without membrane penetration. To evaluate this theory, tracking the localization of fluorescently tagged PE_PGRS1 during host cell infection could reveal whether endocytosis of foci occurs. Analysis of the ESX-dependent proteins EspB shows a significant phase separation propensity as well (Supplemental Figure S3), making comparative analyses of interactions of PE_PGRS proteins and EspB, and chaperones very attractive.

Despite extensive research on biomolecular phase separation over the past decade, the role of proteins with a high propensity for phase separation in cell–cell contact, specifically in pathogenicity, remains poorly understood. In fact, phase separation as a mechanism underlying host–microbe interactions has not been discussed before. PE_PGRS proteins provide a unique opportunity to study the potential of phase separation in biomedical science and host–microbe interactions.

## 6. Future Outlook

In this review, we have discussed many aspects of the structure-function relationships of the PE_PGRS protein family. However, it still remains unclear what specific roles PE_PGRS proteins could play, especially when it comes to the interaction of PE_PGRS proteins with host factors such as the TLR receptors. From a structural perspective, even with advanced AI modeling, it currently seems impossible to identify specific patterns in the PE_PGRS protein domains that could explain specialized functions. Considering that structurally similar proteins are also produced by non-pathogenic mycobacteria, such as *M. smegmatis*, one could argue that the protein family originates from an ancestral protein with an important household function. It could be argued that after the duplication events that occurred in pathogens, such a household function has been maintained in many of these proteins but is now overshadowed by the clear potential of PE_PGRS proteins for pathogenic virulence. However, as illustrated by the peculiar *M. marinum PE_PGRS1* gene, it is easy to imagine that such genes, through simple insertion sequence-mediated translocation events, acquired new functions specialized in virulence, becoming “more equal” (a George Orwell terminology) than others, and potentially taking over less sophisticated functions of other virulence factors. Such functions might have become extinct after these more modern inventions have occurred in evolution. It is therefore important that we also pay a lot of attention to comparative analyses of the function of PE_PGRS proteins in many different mycobacterial species including non-pathogenic strains. In analogy, the discovery of the functions of type VII secretion machinery in *M. smegmatis* and *M. marinum* in conjugal transfer has pointed out that proteins involved in pathogenesis could also have very different functions [92]. We envisage that an increased use of the *M. marinum* zebrafish infection model could lead to rapid advances in understanding specializations in the biological functions of the PE_PGRS gene family. In addition, function experiments could also be undertaken in strains that do not produce any PE_PGRS proteins, such as strains from the *M. avium* complex. Considering that these strains do possess a functional *ESX5* secretion system, it will be of interest to study whether heterologously expressed PE_PGRS proteins are secreted in *M. avium*.

As for possible medical applications of increased knowledge of the PE_PGRS proteins, we envisage that these will not only be in the development of new antibiotic or vaccines that target this protein family. After all, an overarching message of microbiological studies is that microbes can teach us much about the mechanisms underlying the basic functions of the cells of their hosts. Therefore, we believe that further studies of the function of PE_PGRS proteins could also lead to new host-directed therapy strategies. Furthermore, more knowledge on the biophysical properties of PE_PGRS proteins could, for instance, explain their mysterious capacity to travel to subcellular compartments of the host. We highlight for the first time the extremely high propensity of the PGRS domain for phase separation that might be correlated with their special translocation properties. This might make PE_PGRS proteins valuable potential tools that could be used in strategies for designing novel treatments for a variety of disease. Essentially, the potential of using PE_PGRS proteins as a therapeutic tool is dependent on whether combatting the particular disease is mostly benefitting from stimulating hyper-inflammatory or immunosuppressive responses, both of which might be accomplished by employing particular PE_PGRS proteins.

## 7. Conclusions

There are various indications that several representatives of the PE_PGRS proteins evolved specialized functions in recent evolution. The genes encoding these specialized proteins are apparently closely linked to transcriptional control by host factors. We identified a particular representative PE_PGRS gene in *M. marinum* that is linked to control by host sterols. Computational analysis and structural annotation using AlphaFold3 combined with other tools reveals an exceptionally powerful and unprecedented ability to undergo phase separation by the PGRS domain. This suggests that PGRS’s glycine-rich, multivalent, low-complexity composition supports phase separation while adopting a structured conformation, contrary to the disordered nature typical of such domains. The role of PE_PGRS proteins as secreted factors influencing host processes indicates a novel window into the role of phase separation as never before reported, broadening the mechanistic understanding of microbe–host interactions.

## Data Availability

The original contributions presented in this study are included in the article/Supplemental Materials. Further inquiries can be directed to the corresponding author.

## Supplementary Materials

The following supporting information can be downloaded at https://www.mdpi.com/article/10.3390/biology14030247/s1: Figure S1: Neighbor-joining tree of all *M. marinum* PE_PGRS1 homologs in mycobacteria based on NCBI BLAST analysis. Default algorithmic parameters for BLAST analysis were used; Figure S2: AlphaFold3 structural modelling of PE_PGRS homologs; Figure S3: Sequence-based phase separation predictions of EspB proteins using the PLAAC and PScore algorithms; Figure S4: AlphaFold3 model of *M. tuberculosis* PE_PGRS33 and *D. rerio* TLR2, with a protein sequence alignment between PE_PGRS33 and PE_PGRS1; Figure S5: Prediction values of AlphaFold3 models of PE_PGRS1, PE_PGRS1—TLR2, and PE_PGRS33—TLR2; Figure S6: Prediction values of AlphaFold3 models of homologs of PE_PGRS1 shown in Figure S2; Table S1: FASTA table of the protein sequences used to construct the neighbor-joining tree of Figure 1; Table S2: Excel table showing all BLAST hits with the HPS boxes of *M. marinum* and *M. abscessus*.
