# Supplementary material for: Evolution of the PE_PGRS Proteins of Mycobacteria: Are All Equal or Are Some More Equal than Others?
Source: Biology (Basel). 2025 Feb 28;14(3):247. doi: 10.3390/biology14030247 (PMC11939664; doi:10.3390/biology14030247)
Supplement: Supplementary file 1 [file biology-14-00247-s001.zip › Supplemental figure 1.pdf]

Figure S1: Neighbor joining tree of all *M. marinum* PE_PGRS1 homologs in mycobacteria based on NCBI BLAST analysis. Default algorithmic parameter for BLAST analysis were used. We used fast minimum evolution settings at NCBI: https://blast.ncbi.nlm.nih.gov/Blast.cgi

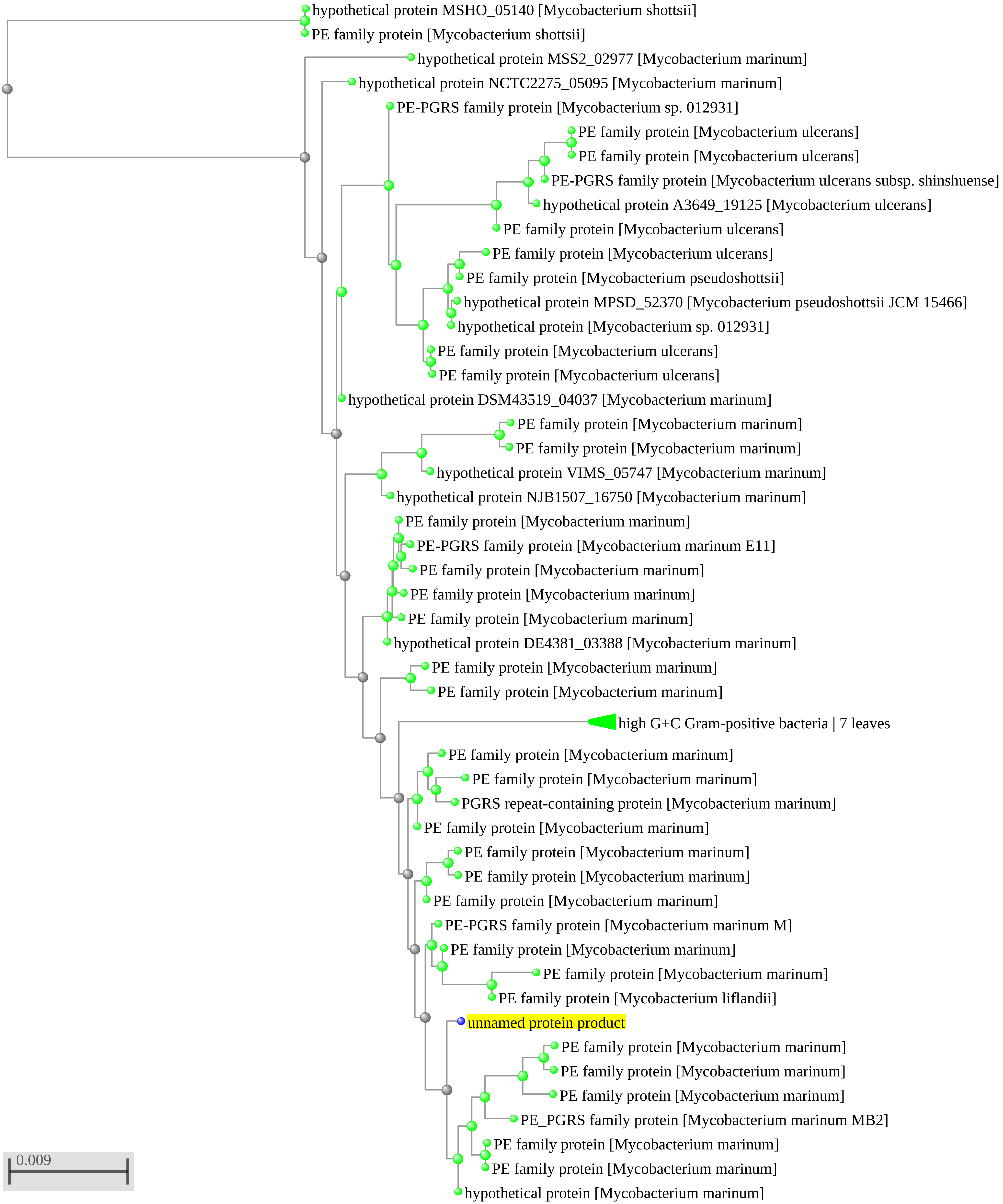
hypothetical protein MSHO_05140 [Mycobacterium shottsii]
PE family protein [Mycobacterium shottsii]
hypothetical protein MSS2_02977 [Mycobacterium marinum]
hypothetical protein NCTC2275_05095 [Mycobacterium marinum]
PE-PGRS family protein [Mycobacterium sp. 012931]
PE family protein [Mycobacterium ulcerans]
PE family protein [Mycobacterium ulcerans]
PE-PGRS family protein [Mycobacterium ulcerans subsp. shinshuense]
hypothetical protein A3649_19125 [Mycobacterium ulcerans]
PE family protein [Mycobacterium ulcerans]
PE family protein [Mycobacterium ulcerans]
PE family protein [Mycobacterium pseudoshottsii]
hypothetical protein MPSD_52370 [Mycobacterium pseudoshottsii JCM 15466]
hypothetical protein [Mycobacterium sp. 012931]
PE family protein [Mycobacterium ulcerans]
PE family protein [Mycobacterium ulcerans]
hypothetical protein DSM43519_04037 [Mycobacterium marinum]
PE family protein [Mycobacterium marinum]
PE family protein [Mycobacterium marinum]
hypothetical protein VIMS_05747 [Mycobacterium marinum]
hypothetical protein NJB1507_16750 [Mycobacterium marinum]
PE family protein [Mycobacterium marinum]
PE-PGRS family protein [Mycobacterium marinum E11]
PE family protein [Mycobacterium marinum]
PE family protein [Mycobacterium marinum]
PE family protein [Mycobacterium marinum]
hypothetical protein DE4381_03388 [Mycobacterium marinum]
PE family protein [Mycobacterium marinum]
PE family protein [Mycobacterium marinum]
high G+C Gram-positive bacteria | 7 leaves
PE family protein [Mycobacterium marinum]
PE family protein [Mycobacterium marinum]
PGRS repeat-containing protein [Mycobacterium marinum]
PE family protein [Mycobacterium marinum]
PE family protein [Mycobacterium marinum]
PE family protein [Mycobacterium marinum]
PE family protein [Mycobacterium marinum]
PE-PGRS family protein [Mycobacterium marinum M]
PE family protein [Mycobacterium marinum]
PE family protein [Mycobacterium marinum]
PE family protein [Mycobacterium liflandii]
unnamed protein product
PE family protein [Mycobacterium marinum]
PE family protein [Mycobacterium marinum]
PE family protein [Mycobacterium marinum]
PE_PGRS family protein [Mycobacterium marinum MB2]
PE family protein [Mycobacterium marinum]
PE family protein [Mycobacterium marinum]
hypothetical protein [Mycobacterium marinum]
0.009
