# Supplementary material for: Evolution of the PE_PGRS Proteins of Mycobacteria: Are All Equal or Are Some More Equal than Others?
Source: Biology (Basel). 2025 Feb 28;14(3):247. doi: 10.3390/biology14030247 (PMC11939664; doi:10.3390/biology14030247)
Supplement: Supplementary file 1 [file biology-14-00247-s001.zip › Supplemental Figure 2.pdf]

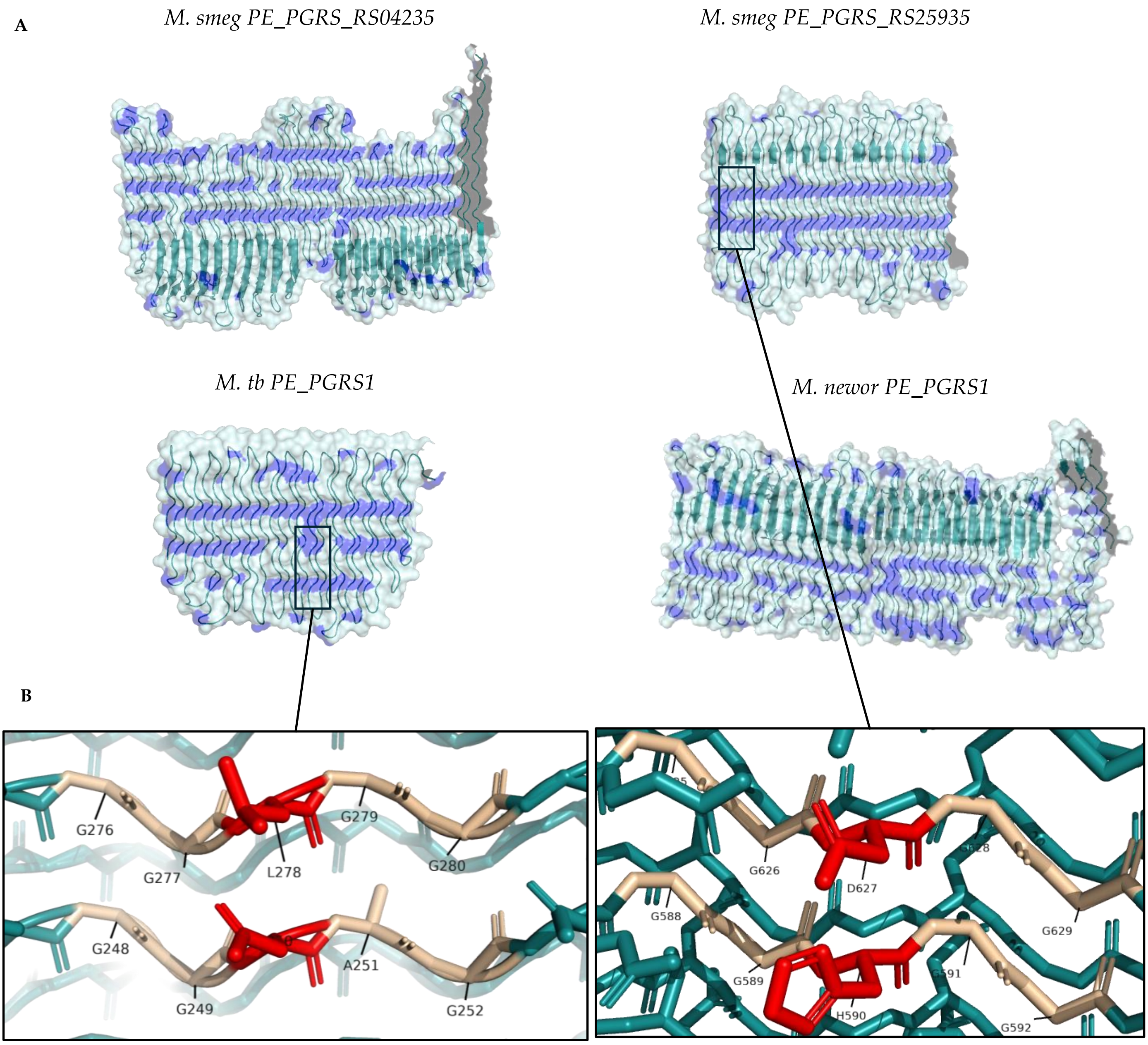

Supplemental Figure 2. Predicted structures of representative members of the PE_PGRS family in *M. smegmatis, M. tuberculosis,* and *M. neworleansis*. PE regions were cut off due to low quality prediction values as visible in Supplemental Figure 6.
