# Supplementary material for: Evolution of the PE_PGRS Proteins of Mycobacteria: Are All Equal or Are Some More Equal than Others?
Source: Biology (Basel). 2025 Feb 28;14(3):247. doi: 10.3390/biology14030247 (PMC11939664; doi:10.3390/biology14030247)
Supplement: Supplementary file 1 [file biology-14-00247-s001.zip › Supplemental Figure 3.pdf]

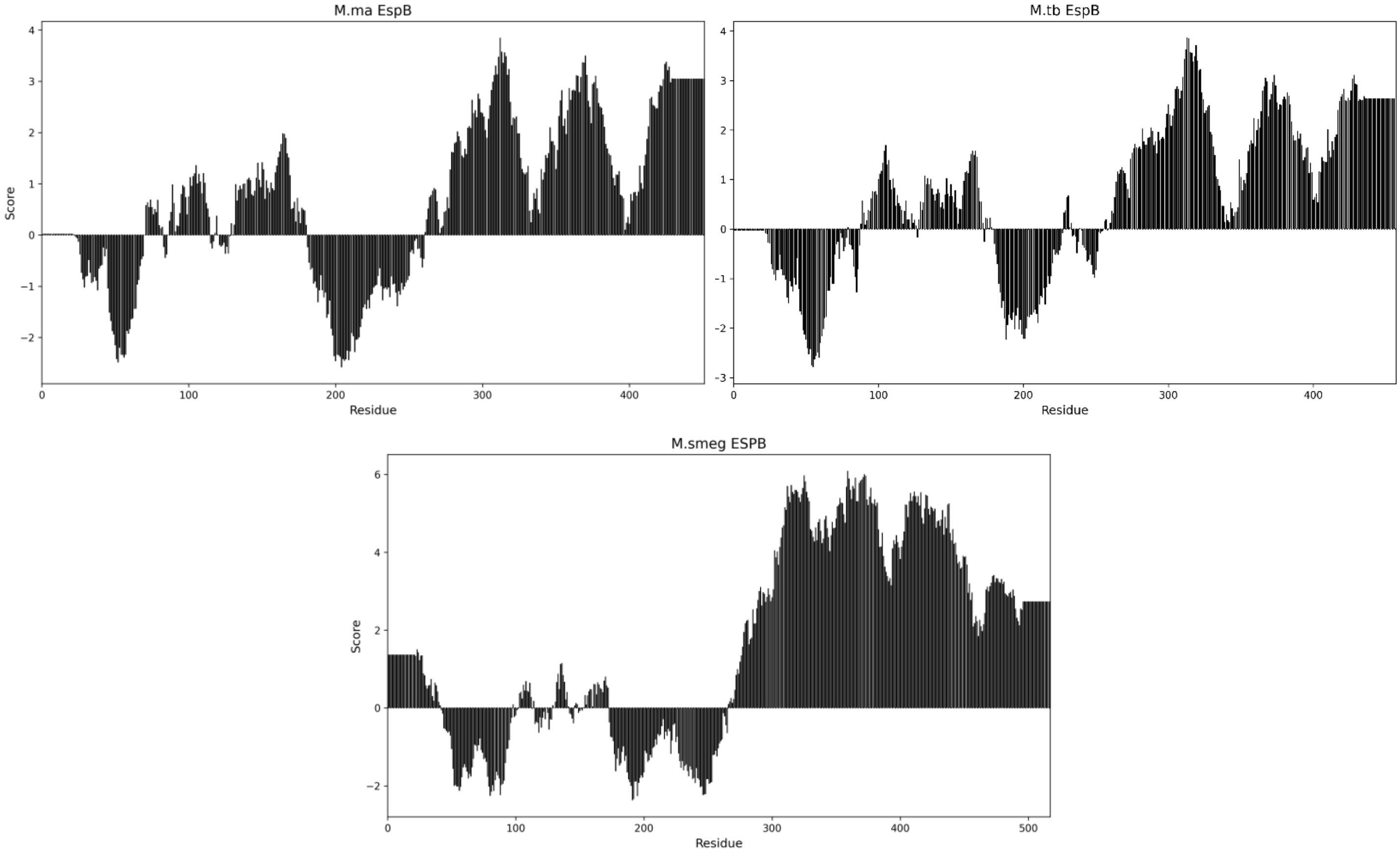

Supplemental Figure 3. PScore calculation plotted using python for *M. marinum* EspB, *M tuberculosis* EspB, and *M. smegmatis* EspB.
