# Supplementary material for: Evolution of the PE_PGRS Proteins of Mycobacteria: Are All Equal or Are Some More Equal than Others?
Source: Biology (Basel). 2025 Feb 28;14(3):247. doi: 10.3390/biology14030247 (PMC11939664; doi:10.3390/biology14030247)
Supplement: Supplementary file 1 [file biology-14-00247-s001.zip › Supplemental Figure 4.pdf]

A

M. tuberculosis PE_PGRS33 – D. rerio TLR2

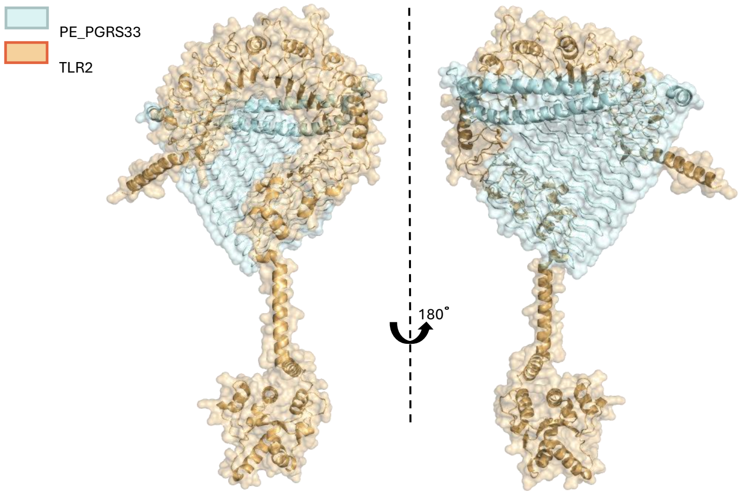

B

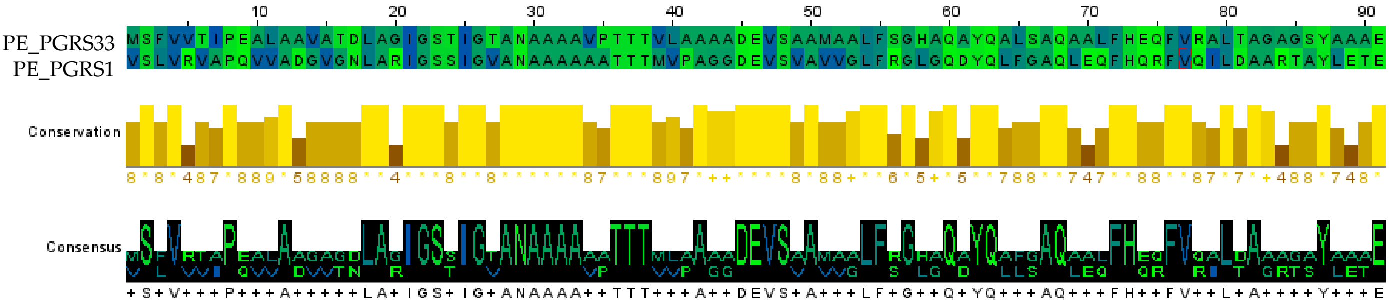

Supplemental Figure 4. Panel 4A: Predicted interaction between *M. tuberculosis* PE_PGRS33 and *D. rerio* TLR2, made using AlphaFold3 on AlphaFoldServer and visualized using PyMol. PE_PGRS33 is colored light orange, and TLR2 in pale cyan. Panel 4B: Protein sequence alignment of *M. smegmatis* PE_PGRS33 and *M. marinum* PE_PGRS1, with residues coloured by buried index, created with NCBI Blast and visualized in Jalview.
