# Supplementary material for: Evolution of the PE_PGRS Proteins of Mycobacteria: Are All Equal or Are Some More Equal than Others?
Source: Biology (Basel). 2025 Feb 28;14(3):247. doi: 10.3390/biology14030247 (PMC11939664; doi:10.3390/biology14030247)
Supplement: Supplementary file 1 [file biology-14-00247-s001.zip › Supplemental Figure 6.pdf]

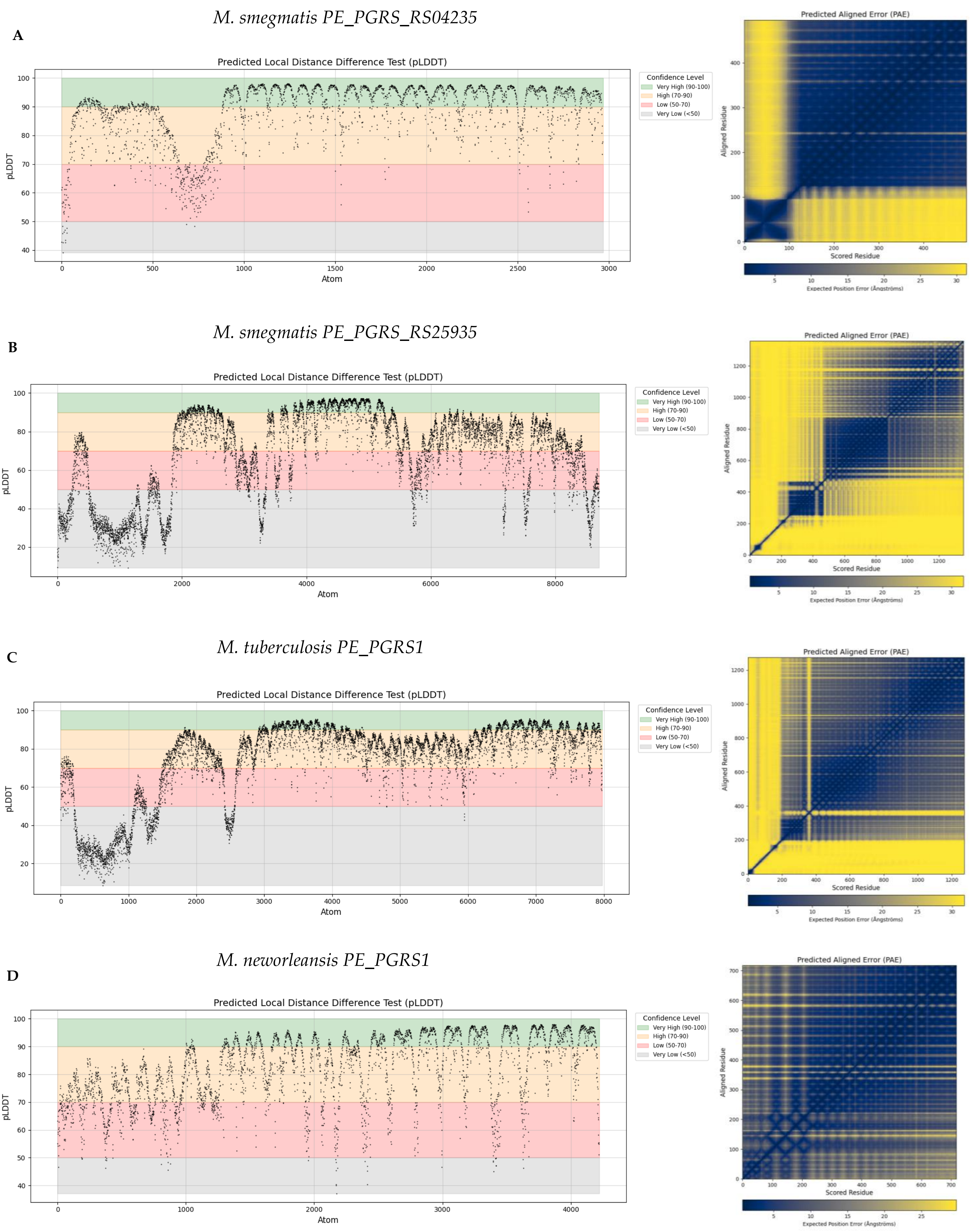

Supplemental Figure 6. Prediction values of the AlphaFold3 predictions visualized using the pLDDT

and PAE scores with af_plotter.
